# Supplementary material for: Chemical Composition and Insecticidal Activities of Essential Oils against the Pulse Beetle
Source: Molecules. 2022 Jan 17;27(2):568. doi: 10.3390/molecules27020568 (PMC8777654; doi:10.3390/molecules27020568)
Supplement: Supplementary file 1 [file molecules-27-00568-s001.zip › molecules-1495441-supplementary.pdf]

Chemical Composition and Insecticidal Activities of Essential Oils against the Pulse Beetle

C. S. Jayaram [1], Nandita Chauhan [1], Shudh Kirti Dolma [1,2], S. G. Eswara Reddy [1,2]*

[1]Entomology Laboratory, Agrotechnology Division, CSIR-Institute of Himalayan Bioresource Technology, Palampur-176061, Himachal Pradesh (INDIA)

[2]Academy of Scientific and Innovative Research (AcSIR), Ghaziabad- 201 002, Uttar Pradesh, India

*Correspondence:ereddy2001@yahoo.com, ereddy@ihbt.res.in (S.G. Eswara Reddy)

Supplementary material

Sr. No.	Name	RI[a]	RI[b]	Area%	Mode of Identification
1	*α*-pinene	939	939	0.65	MS, RI
2	Sabinene	976	976	0.33	MS, RI
3	*β*-pinene	980	982	1.03	MS, RI
4	*β*-phellandrene	1031	1028	0.46	MS, RI
5	Limonene	1031	1033	2.43	MS, RI
6	1,8-cineole	1033	1037	6.31	MS, RI
7	*γ*-terpinene	1062	1061	0.17	MS, RI
8	*cis*-sabinene hydrate	1068	1073	0.24	MS, RI
9	Menthone	1154	1161	29.54	MS, RI
10	Menthofuran	1164	1168	6.49	MS, RI
11	Menthol	1173	1173	0.63	MS, RI
12	*neo*-isomenthol	1188	1184	38.64	MS, RI
13	*α*-terpineol	1189	1196	0.40	MS, RI
14	Carvone	1242	1240	0.66	MS, RI
15	Piperitone	1252	1256	0.37	MS, RI
16	*neo*-menthyl acetate	1275	1273	0.23	MS, RI
17	Menthyl acetate	1294	1290	7.55	MS, RI
18	Isomenthyl acetate	1306	1304	0.11	MS, RI
19	*β*-caryophyllene	1418	1421	2.06	MS, RI
20	*β*-farnesene	1443	1439	0.33	MS, RI
21	Germacrene-D	1480	1482	0.10	MS, RI
22	*β*-himachalene	1499	1500	0.44	MS, RI
23	Caryophyllene oxide	1581	1583	0.41	MS, RI
24	-	-	1914	0.18	MS, RI
25	-	-	1924	0.2	MS, RI
	Total			100.0	
	Monoterpene hydrocarbons*			5.07	
	Oxygenated monoterpene*			83.28	
	Sesquiterpene hydrocarbons*			2.93	
	Oxygenated sesquiterpene*			0.41	

[a]Retention index value of compounds in the literature (Adams 2007).
[b]Retention index value determined relative to *n*-alkanes (C9-C24) on the DB-5 GC column.
*Percentage of compounds class in analyzed essential oil samples.
MI=Mode of Identification

Table S1. The chemical composition of essential oil of *Mentha piperita*

Sr. No.	Name	RI[a]	RI[b]	Area%	Mode of Identification
1	*α*-pinene	939	939	1.01	MS, RI
2	Sabinene	976	976	0.53	MS, RI
3	*β*-pinene	980	982	0.93	MS, RI
4	*β*-myrcene	991	990	1.83	MS, RI
5	*β*-phellandrene	1031	1028	0.33	MS, RI
6	Limonene	1031	1034	21.30	MS, RI
7	1,8-cineole	1033	1037	2.29	MS, RI
8	*γ*-terpinene	1062	1061	0.23	MS, RI
9	*cis*-sabinene hydrate	1068	1074	0.29	MS, RI
10	Menthone	1154	1158	0.52	MS, RI
11	4-terpineol	1177	1182	0.76	MS, RI
12	*α*-terpineol	1189	1195	0.22	MS, RI
13	*cis*-dihydrocarvone	1193	1197	1.40	MS, RI
14	*trans*-dihydrocarvone	1200	1204	0.21	MS, RI
15	Carvone	1242	1251	63.38	MS, RI
16	Piperitone	1252	1257	0.33	MS, RI
17	*iso*-dihydro carvyl acetate	1325	1324	0.22	MS, RI
18	*cis*-carvyl acetate	1362	1359	0.22	MS, RI
19	*β*-bourbonene	1384	1385	1.25	MS, RI
20	*β*-caryophyllene	1418	1421	0.98	MS, RI
21	*β*-farnesene	1443	1435	0.17	MS, RI
22	*α*-humulene	1454	1452	0.52	MS, RI
23	Germacrene-D	1480	1482	0.56	MS, RI
24	-	-	1914	0.22	MS, RI
25	-	-	1924	0.30	MS, RI
	Total			99.48	
	Monoterpene hydrocarbons*			26.16	
	Oxygenated monoterpene*			69.40	
	Sesquiterpene hydrocarbons*			3.48	
	Oxygenated sesquiterpene*			0	

[a]Retention index value of compounds in the literature (Adams 2007).
[b]Retention index value determined relative to *n*-alkanes (C9-C24) on the DB-5 GC column.
*Percentage of compounds class in analyzed essential oil samples.
MI=Mode of Identification

Table S2. The chemical composition of essential oil of *Mentha spicata*

Sr. No.	Name	RI[a]	RI[b]	Area%	Mode of Identification
1	Limonene	1031	1033	3.60	MS, RI
2	*β*-ocimene	1040	1040	40.57	MS, RI
3	Dihydrotagetone	1054	1055	28.74	MS, RI
4	*Z*-ocimene	1128	1130	1.11	MS, RI
5	*E*-tagetone	1146	1147	0.71	MS, RI
6	*Z*-tagetone	1153	1155	11.63	MS, RI
7	*Z*-ocimenone	1231	1233	3.42	MS, RI
8	*E*-ocimenone	1239	1241	8.72	MS, RI
9	*β*-caryophyllene	1418	1422	0.73	MS, RI
10	Bicyclogermacrene	1494	1497	0.76	MS, RI
	Total			100.0	
	Monoterpene hydrocarbons*			45.28	
	Oxygenated monoterpene*			53.22	
	Sesquiterpene hydrocarbons*			1.49	
	Oxygenated sesquiterpene*			0.00	

[a]Retention index value of compounds in the literature (Adams 2007).
[b]Retention index value determined relative to *n*-alkanes (C9-C24) on the DB-5 GC column.
*Percentage of compounds class in analyzed essential oil samples.
MI=Mode of Identification

Table S3. The chemical composition of essential oil of *Tagetes minuta*

**Conc. (µL/mL)**	**% Repellency; Hours after treatment (*Mean ± SD)**			
	Mentha piperita			
	1 h	2 h	3 h	4 h
8	92±4.89a	88±4.89a	92±4.89a	92±4.89a
6	72±10.19b	72±10.19b	68±10.19b	68±10.19a
4	44±7.48c	36±11.66c	28±20.59c	24±23.15b
2	32±13.56c	28±8.00c	16±4.00c	16±9.79b
1	12±8.00c	12±8.00c	12±4.89c	8±4.89b
	$F_{4,24}$=11.72; p<0.0001	$F_{4,24}$=12.79; p<0.0001	$F_{4,24}$=10.43; p<0.0001	$F_{4,24}$=8.50; p<0.0001
	Mentha spicata			
8	76±7.48a	80±8.94a	80±8.94a	84±9.79a
6	60±6.32ab	64±4.00b	64±4.00ab	72±4.89b
4	44±9.79bc	40±6.32c	40±6.32b	40±14.14c
2	40±10.95c	36±7.48cd	36±9.79c	32±12.00c
1	16±4.00c	12±4.89d	8±4.89c	8±4.89c
	$F_{4,24}$=7.73; p<0.001	$F_{4,24}$=16.03; p<0.0001	$F_{4,24}$=14.93 p<0.0001	$F_{4,24}$=9.70; p<0.001
	Tagetes minuta			
8	76±7.48a	84±11.66a	96±4.00a	96±4.00a
6	64±4.00ab	60±14.14ab	60±8.94b	60±11.66b
4	56±9.79ab	52±17.43ab	36±11.66c	36±9.79c
2	44±11.66b	36±7.48b	28±4.89c	28±4.89c
1	36±7.48b	26±7.60b	16±4.00c	16±4.89c
	$F_{4,24}$=3.49; p<0.026	$F_{4,24}$=3.29; p<0.032	$F_{4,24}$=18.44; p<0.0001	$F_{4,24}$=23.03; p<0.0001

*Mean of 5 replications; Means followed by the same letters within a column do not differ significantly by Tukey's HSD ($P \leq 0.05$).

Table S4. Repellency of essential oils against *Callosobruchus chinensis*

Conc. (µL/mL)	% Repellency; Hours after treatment (*Mean ± SD)			
	**1 h**	**2 h**	**3 h**	**4 h**
	Mentha spicata			
8	76±11.66a	76±7.48a	52±4.89a	48.00±4.90a
6	52±10.19ab	56±4.00b	24±7.48b	24.00±7.48b
4	36±7.48bc	40±6.32c	16±4.00b	12.00±4.90b
2	24±9.79c	20±8.94c	12±4.89b	8.00±4.90b
1	8±4.89c	16±7.48c	8±4.89b	4.00±4.00b
	$F_{4,24}$=8.21; p<0.0001	$F_{4,24}$=12.67; p<0.0001	$F_{4,24}$=10.72; p<0.0001	$F_{4,24}$=10.94; p<0.0001
	Tagetes minuta			
8	64±7.48a	76±7.48a	52±8.00	51±4.89a
6	52±13.56ab	40±8.94b	32±4.89	40±6.32ab
4	40±8.94b	32±10.19b	28±14.96	24±9.79b
2	24±11.66b	24±11.66b	24±7.48	20±6.32b
1	20±8.94b	20±8.94b	20±8.94	16±7.48b
	$F_{4,24}$=3.20; p<0.035	$F_{4,24}$=5.49; p<0.004	$F_{4,24}$=1.73; p>0.18	$F_{4,24}$=4.33; p<0.01
	Mentha piperita			
8	80.00±6.32a	72.00±4.90a	80.00±6.32a	76.00±7.48a
6	64.00±13.27ab	60.00±6.32ab	56.00±7.48ab	64.00±11.66ab
4	64.00±7.48ab	40.00±6.32bc	48.00±4.90bc	52.00±10.20ab
2	36.00±7.48c	32.00±10.20bc	40.00±10.95c	36.00±7.48bc
1	24.00±4.00c	20.00±8.94c	16.00±7.48c	12.00±4.90c
	$F_{4,24}$=7.63; p<0.001	$F_{4,24}$=7.69; p<0.001	$F_{4,24}$=9.19; p<0.0001	$F_{4,24}$=8.30; p<0.0001

*Mean of 5 replications; Means followed by the same letters within a column do not differ significantly by Tukey's HSD ($P \leq 0.05$).

Table S5. Repellency of essential oils against *Callosobruchus maculatus*

**Conc. (μL/mL)**	**% Inhibition; Hours after treatment (*Mean ± SD)**		
	**24 h**	**48 h**	**72 h**
	Mentha piperita		
20	100.00±0.00a	96.00±1.30a	90.80±1.50 a
10	85.40±2.54b	79.20±1.80b	77.00±1.48b
5	76.60±3.41b	72.00±1.84b	75.00±2.51b
2.5	52.80±3.60c	56.00±3.48c	60.20±3.31c
1.25	26.00±5.47d	34.20±2.75d	39.40±3.23d
	$F_{4, 24}$=88.50; P<0.0001	$F_{4, 24}$=98.76; P<0.0001	$F_{4, 24}$=59.41; P<0.0001
	Mentha spicata		
10	100.00±0.00a	95.40±1.40a	92.00±1.14a
8	98.00±2.00a	87.60±2.38ab	82.20±1.59ab
6	79.40±3.01b	76.20±5.11bc	67.40±8.13b
4	63.80±5.23c	62.60±3.26c	65.60±5.09b
2	38.40±4.58d	24.00±5.52d	20.60±4.02c
	$F_{4, 24}$=53.66; P<0.0001	$F_{4, 24}$=52.73; P<0.0001	$F_{4, 24}$=33.49; P<0.0001
	Tagetes minuta		
10	100.00±0.00a	100.00±0.00a	99.40±0.60a
8	100.00±0.00a	93.60±2.58a	93.20±1.28a
6	85.80±5.31ab	77.80±4.02b	79.40±2.46b
4	52.80±15.82bc	46.20±5.54c	51.40±2.73c
2	30.40±5.27c	36.00±2.35c	36.40±2.25d
	$F_{4, 24}$=15.68; P<0.0001	$F_{4, 24}$=68.66; P<0.0001	$F_{4, 24}$=178.98; P<0.0001

*Mean of 5 replications; Means followed by the same letters within a column do not differ significantly by Tukey's HSD ($P \leq 0.05$).

Table S6. Ovipositional inhibition of essential oils against *Callosobruchus chinensis*

**Conc. (µL/mL)**	**% Inhibition; Hours after treatment (*Mean ± SD)**		
	**24 h**	**48 h**	**72 h**
	Mentha piperita		
12	100.00±0.00a	94.66±3.44a	77.60±2.51a
8	86.36±2.25a	87.26±1.59a	75.78±2.74a
4	62.54±4.06b	66.64±5.25b	58.26±6.46b
2	41.28±3.90b	44.58±2.41c	52.04±2.21b
1	15.28±5.28c	23.5±3.56d	28.24±0.69c
	$F_{4, 24}$=89.86; *P*<0.0001	$F_{4, 24}$=72.46; *P*<0.0001	$F_{4, 24}$=33.26; *P*<0.0001
	Mentha spicata		
12	94.26±3.66a	91.06±1.89a	75.12±3.32a
8	67.42±4.46b	71.88±3.85b	50.66±4.55b
4	52.6±1.68b	50.78±2.95c	35.78±2.71c
2	29.02±6.11c	27.91±5.66d	21.90±4.93d
1	18.44±4.77c	9.91±2.71e	8.24±2.12e
	$F_{4, 24}$=47.75; *P*<0.0001	$F_{4, 24}$=80.04; *P*<0.0001	$F_{4, 24}$=49.54; *P*<0.0001
	Tagetes minuta		
12	44.96±4.01	37.24±5.16a	35.68±4.09a
8	43.60±5.63	35.72±5.87ab	30.64±6.91ab
4	39.88±3.90	22.82±3.09bc	19.84±3.51ab
2	33.88±3.36	18.29±4.75c	17.14±1.97c
1	28.12±6.30	16.68±2.44c	14.15±2.24c
	$F_{4, 24}$=2.17; *P*>0.1	$F_{4, 24}$=4.75; *P*<0.007	$F_{4, 24}$=4.96; *P*<0.006

*Mean of 5 replications; Means followed by the same letters within a column do not differ significantly by Tukey's HSD ($P \leq 0.05$).

Table S7. Ovipositional inhibition of essential oils against *Callosobruchus maculatus*
